# Clinical Characteristics of Peripheral Neuropathy in Eosinophilic Granulomatosis with Polyangiitis: A Retrospective Single-Center Study in China

**DOI:** 10.1155/2020/3530768

**Published:** 2020-07-04

**Authors:** Zhaocui Zhang, Suying Liu, Ling Guo, Li Wang, Qingjun Wu, Wenjie Zheng, Yong Hou, Xinping Tian, Xiaofeng Zeng, Fengchun Zhang

**Affiliations:** ^1^Department of Rheumatology and Clinical Immunology, Peking Union Medical College Hospital, Chinese Academy of Medical Sciences & Peking Union Medical College, The Ministry of Education Key Laboratory, National Clinical Research Center for Dermatologic and Immunologic Diseases, Beijing 100730, China; ^2^Department of Rheumatology and Clinical Immunology, Gansu Province People's Hospital, Lanzhou, Gansu Province 730000, China; ^3^Department of Rheumatology, Dongying People's Hospital, Dongying, Shandong Province 257000, China

## Abstract

**Objective:**

To investigate clinical features, independent associated factors, treatment, and outcome of patients with peripheral neuropathy (PN) in eosinophilic granulomatosis with polyangiitis (EGPA).

**Methods:**

We retrospectively analyzed clinical data of 110 EGPA patients from 2007 to 2019 in Peking Union Medical College Hospital. The independent factors associated with PN in EGPA were analyzed with univariate and multivariate logistic regressions.

**Results:**

In EGPA with PN, paresthesia and muscle weakness were observed in 82% and 33% of patients, respectively. Both the upper and lower limbs were involved in 51% of patients. 30% of EGPA patients had symmetrical multiple peripheral neuropathy, whereas only 16.4% presented with mononeuritis multiplex. Compared to patients without PN, patients with PN had a higher erythrocyte sedimentation rate, C-reactive protein, rheumatoid factor, Birmingham vasculitis activity score (BVAS), and positivity of myeloperoxidase-antineutrophil cytoplasmic antibodies (MPO-ANCA). Regarding manifestations, patients with PN tended to develop weight loss and arthritis or joint pain. Notably, ANCA positivity, arthritis or joint pain, and higher BVAS were found to be independent associated factors for PN in EGPA. Patients with PN more frequently need glucocorticoid pulses and intravenous infusion of cyclophosphamide. With the longest follow-up of 11.0 years, we found that age and cardiac involvement were risk factors for survival, and female was the protective factor.

**Conclusion:**

PN in EGPA frequently displays with symmetrical multiple peripheral neuropathy in China. Positive ANCA, arthritis or joint pain, and higher BVAS are the independent associated factors of PN in EGPA. Glucocorticoids with immunosuppressants are vital therapeutic strategy.

## 1. Introduction

Eosinophilic granulomatosis with polyangiitis (EGPA), formerly called Churg-Strauss Syndrome, is an antineutrophil cytoplasmic antibody- (ANCA-) associated systemic necrotizing small-vessel vasculitis (AAV) [1]. Hallmarks of EGPA include asthma, hypereosinophilic syndrome, extravascular granuloma, and life-threatening vasculitis, possibly affecting the lung, heart, peripheral nerves, kidney, and other important organs [2–5]. Although EGPA is a form of AAV, ANCA only exists in approximately one-third of EGPA patients, which always displays with a perinuclear labeling on immunofluorescence analysis with specificity against myeloperoxidase (MPO) [1, 6, 7].The disease course of classical EGPA is roughly divided into three phases. The first phase is prodromal phase, which is manifested as allergic symptoms such as asthma, sinusitis, nasal polyps, or allergic rhinitis. This phase can last for several decades. The second phase is dominated by tissue eosinophilia, and the last phase is characterized by vasculitis, most commonly involving peripheral nerves, skin, and kidneys [1].

Peripheral neuropathy (PN) is a prevalent and important manifestation of EGPA [8, 9] which has a very negative impact on life quality of the patients. Additionally, it is difficult to connect manifestation of PN with the diagnosis of EGPA when PN is the initial symptom. Samson et al. found that mononeuritis multiplex predicted the need for immunomodulatory drugs for EGPA, which indicated that sometimes PN in EGPA signified intensive treatment [10]. Therefore, for early diagnosis and intervention of PN in EGPA, it is of great significance to thoroughly review the features of PN in EGPA.

Based on data from 110 EGPA patients from our institution, we investigated the clinical features, treatment, and outcome of EGPA with PN and explored independent associated factors in order to deepen clinicians' insights into EGPA with PN.

## 2. Patients and Methods

### 2.1. Patients

We retrospectively analyzed 110 EGPA patients admitted to Peking Union Medical College Hospital (PUMCH) between January 2007 and March 2019. All patients fulfilled the criteria of the 2012 Revised International Chapel Hill Consensus Conference Nomenclature for Vasculitides [11] and were verified by two rheumatologists. The diagnosis of PN was based on clinical manifestations of the nervous system, electromyography, or neuropathology and confirmed by at least one neurologist. Because the study was based on a review of medical records which had been acquired for clinical purposes, the requirement for written informed consent was waived. The local institutional review board approved the study.

### 2.2. Clinical and Laboratory Assessment

Mononeuritis multiplex was defined as “simultaneously or successively involving two or more separate, nonadjacent nerve trunks”. Multiple peripheral neuropathy was defined as “a bilaterally symmetric, widely distributed peripheral neuropathy that predominantly affected distal extremities”. Cutaneous vasculitis included palpable purpura, reticulata, and gangrene ischemia of extremities. Renal involvement was defined as abnormal urine test (hematuria and/or tubular urine and/or quantitation of urine protein more than 0.5g/24 hours) and/or serum creatinine beyond upper limit of normal range. Digestive system involvement was defined as gastrointestinal bleeding, intestinal obstruction, or other findings that could not be explained by other mechanisms. Central nervous system (CNS) involvement was defined as headache, intracranial ischemia, aseptic meningitis, or other findings that could not be explained by other mechanisms. The subacute and chronic onset were defined as less than one month and more than one month, respectively. The original 1994 Birmingham vasculitis activity score (BVAS) [12] was used to assess the disease activity at diagnosis. Patients' outcome included complete remission, partial relief , and all-cause death. Complete remission was defined as a BVAS = 0 and partial relief as a ≥50% reduction in BVAS compared with baseline. According to the 2009 revised five-factor score (FFS) system [13], each patient was scored for the prognosis assessment.

### 2.3. Statistical Analysis

Statistical analysis was performed by SPSS (version 25; IBM, Armonk, NY, USA). Kolmogorov-Smirnova tests were used to assess the normal distribution. Data of normal distribution and non-normal distribution were expressed as the mean ± standard deviation (SD), and median and interquartile range (IQR), respectively. Continuous normal distribution data were compared with the independent samples *t*-tests. Continuous non-normally distributed data was assessed by nonparametric tests. Categorical variables were analyzed by Fisher's exact tests or chi-squared tests. Kaplan-Meier survival curves and log-rank tests were used to compare the survival rate. Univariate and multivariate logistic regression analyses were performed to estimate independent associated factors for PN in EGPA. Receiver operating characteristic (ROC) curve was used to determine the cutoff value of BVAS for PN in EGPA according to the Area Under Curve (AUC) , sensitivity and specificity. *P* values < 0.05 were considered statistically significant.

## 3. Results

### 3.1. Demographics

We retrospectively enrolled 110 EGPA patients consisting of 51 (46.4%) patients with PN and 59 (53.6%) cases without PN. In the patients with PN, mean age of EGPA onset was (47 ± 12.9) (range 23-80) years without gender preference (male to female, 27/24). The average time from initial symptoms to EGPA diagnosis, from allergic symptoms to EGPA diagnosis, and the disease duration was 12 (0, 48) months, 24 (3, 84) months, and 7 (2, 19) months, respectively. PN was observed as an initial manifestation in 5 EGPA patients. These patients came from all over the country.

### 3.2. Clinical and Pathological Features of PN

Table 1 shows the characteristics of PN in EGPA. Among 51 patients, paresthesia (42 cases, 82%) is more common than motor abnormalities (17 cases, 33%); chronic onset was more frequently observed than subacute onset (69% vs. 31%). For the lesion distribution, most cases involved both upper and lower limbs (51%), followed by only lower limb involvement (43%) and only upper limb involvement (6%).

**Figure 1:**
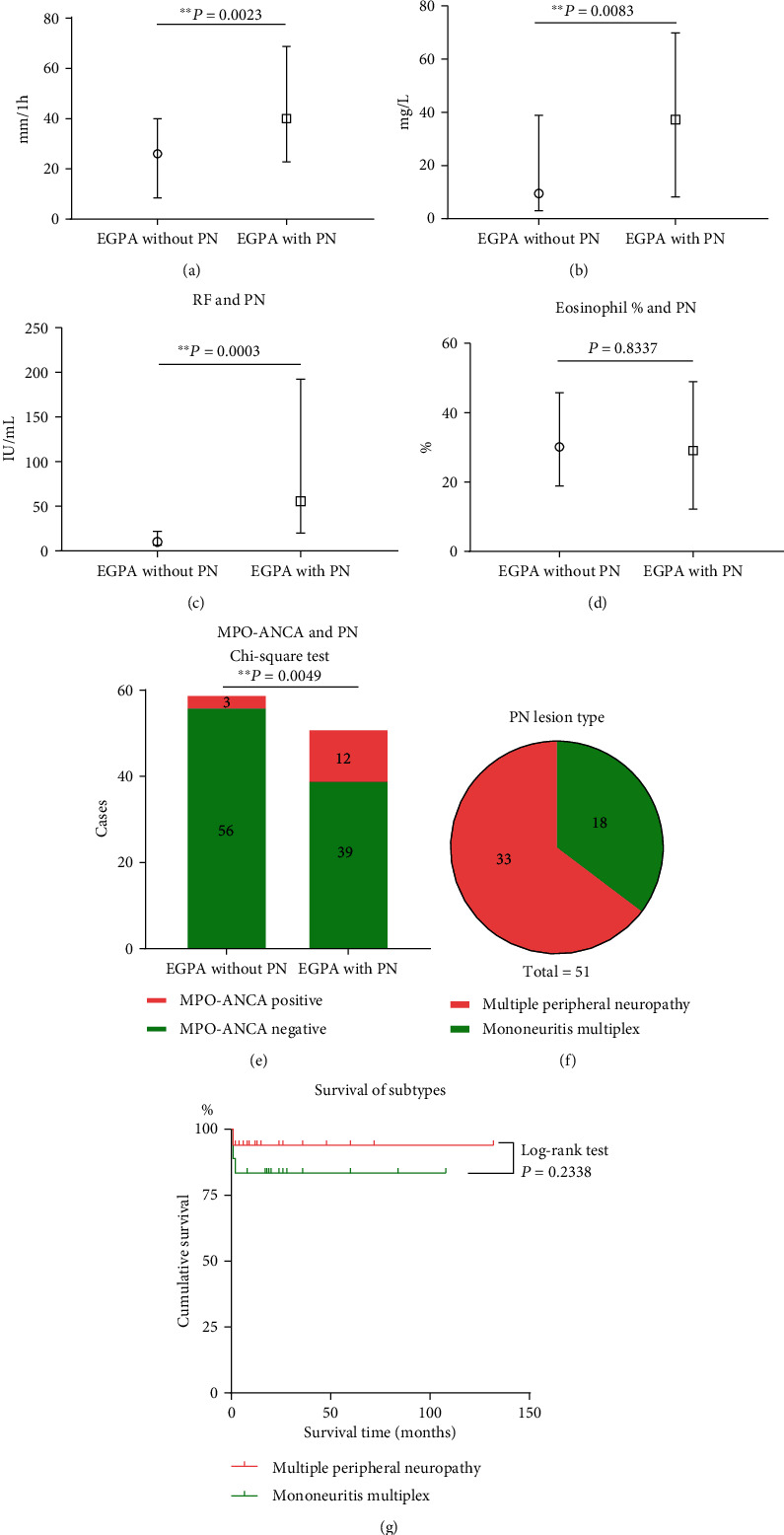
Characteristics of laboratory tests and lesion pattern in EGPA with PN. (a-e) Comparison about serological features between patients with PN and without PN; (f-g) lesion type of PN in EGPA and cumulative survival analysis of the two subgroups; (a-d) Mann–Whitney test was used to compare the rank. PN: peripheral neuropathy; ESR: erythrocyte sedimentation rate; CRP: C-reactive protein; RF: rheumatoid factor; MPO: myeloperoxidase; ANCA: anti-neutrophil cytoplasmic antibody.

Notably, the lesion type of PN in EGPA showed 33 (30%) patients manifested as distal symmetric peripheral neuropathy which was also called multiple peripheral neuropathy, and 18 (16.4%) patients had asymmetrical onset, which displayed with mononeuritis multiplex (Figure 1(f)). Twenty-eight patients underwent electromyography examination, among whom 24 cases were reported to have neurogenic damage, and two patients were reported to have both neurogenic and myogenic damage. The other two patients had no specific neurogenic or myogenic damage despite limb numbness.

Additionally, two patients had a sural nerve biopsy. The pathological features of one patient were acute, severe axonal damage with small-vessel inflammation. The other patient presented with moderately active axonal peripheral neuropathy (primarily a mild-to-moderate decrease in myelinated nerve fiber density) with a small amount of myelinated nerve fiber axonal degeneration and infiltrated by numerous inflammatory cells, which was prominent in cellular immune-mediated peripheral neuropathy.

### 3.3. Systemic Manifestations of EGPA

Among the 51 patients with PN, 40 (78.4%) of the total had asthma, 29 (56.9%) had sinusitis, 26 (51%) had cutaneous vasculitis, 25 (49%) suffered from weight loss, 24 (47.1%) had fever, 22 (43%) had muscle and/or joint pain, 20 (39.2%) developed digestive tract involvement, 20 (39.2%) developed cardiac involvement, 16 (31.4%) were affected by kidney damage, 15 (29.4%) had allergic rhinitis, 11 (21.6%) had CNS involvement, and 8 (15.7%) had ear lesions.

### 3.4. Baseline Clinical Features of EGPA

We analyzed the possible impact of PN on baseline clinical features (Table 2). Compared with EGPA patients without PN, EGPA patients with PN were more likely to have weight loss (49.0% vs. 27.1%, respectively, *P* = 0.018) and arthritis or joint pain (23.5% vs. 8.5%, respectively, *P* = 0.029). Myalgia seemed to be more common in patients with PN than those without PN (27.5% vs. 13.6%, respectively, *P* = 0.069), although it did not reach the statistical difference. Notably, EGPA patients with PN were significantly associated with higher BVAS (median 18 IQR (14, 22) vs. median 12 IQR (8, 16), *P* < 0.0001). Furthermore, the ratio of patients with FFS ≥ 1 in PN was higher than that without PN (60.8% vs. 42.4%, respectively, *P* = 0.054), and the difference was very close to statistical significance. There were no significant differences in fever, asthma, renal involvement, heart involvement, digestive tract involvement, or CNS lesions between the two groups.

### 3.5. Comparison of the Two Subtypes of PN

Table 3 shows that there were no significant differences in the basic demographic characteristics of patients between the two subtypes of PN. Among clinical characteristics, compared with multiple peripheral neuropathy, mononeuritis multiplex was significantly associated with higher proportion of cutaneous vasculitis (77.78% vs. 36.36%, respectively, *P* = 0.005) and renal involvement (50% vs. 21.21%, respectively, *P* = 0.034).The proportion of FFS ≥ 1 in the multiple peripheral neuropathy group was slightly higher than that in mononeuritis multiplex group, but that did not reach statistical difference (66.67% vs. 50%, *P* = 0.244). Concerning the outcome, there was no significant difference in cumulative survival rates between the two types of PN (Log-rank test, *P* = 0.2338) (Figure 1(g)).

### 3.6. Characteristics of Laboratory Tests

We compared the laboratory tests between the two groups (Figures 1(a)-1(e)) and found that patients with PN had a significantly faster erythrocyte sedimentation rate (ESR) (median 40 mm/1 h IQR (23, 69) vs. 26 mm/1 h IQR (9, 40), respectively, *P* = 0.0023), a higher serum level of C-reactive protein (CRP) (median 37.3 mg/L IQR (8.3, 69.9) vs. 9.5 mg/L IQR (3.0, 38.9), respectively, *P* = 0.0083) and a higher rheumatoid factor (RF) (median 56  IU/mL IQR (20, 192) vs. 10  IU/mL IQR (6, 22), respectively, *P* = 0.0003). MPO-ANCA positive ratio was significantly higher (23.53% vs. 5.08%, respectively, *P* = 0.0049) in patients with PN when compared with patients without PN, while the eosinophil ratio did not show statistical difference (31.398 ± 16.895% vs. 30.615 ± 21.236%, *P* = 0.8337).

### 3.7. Baseline Factors That Predict PN

Multivariate logistic regression analysis (Table 4) revealed that ANCA positivity (OR 4.387, 95% CI 1.030–18.683, *P* = 0.045), arthritis or joint pain (OR 3.807, 95% CI 1.038-13.969, *P* = 0.044), and higher BVAS (OR 1.117, 95% CI 1.025-1.217, *P* = 0.012) were independent factors associated with PN onset in EGPA. Next, we used ROC curves to detect the cut-off value for BVAS predicting PN and found the most suitable value was 15 (AUC=0.782, sensitivity 64.71%, specificity 74.58%, P<0.0001) (Supplementary material, Figure S1).

### 3.8. Factors Affecting Survival Time

The longest follow-up of this study was 11.0 years. By multivariate linear regression analysis, we observed that risk factors for survival time included age at onset (standardized *β* = −0.296, *P* = 0.028) and heart involvement (standardized *β* = −0.396, *P* = 0.002), and the protective factor was “female” (standardized *β* = 0.280, *P* = 0.027). While PN, digestive system involvement, renal involvement, and ANCA positivity did not significantly affect the survival time in this specific model (Table 5).

### 3.9. Treatment

A total of 51 EGPA patients with PN received glucocorticoid(GC) therapy without exception, including initial pulses of methylprednisolone (0.5~1.0 g/d, 3~5 days) in 27/51 (52.9%) cases and high-dose prednisone (1~2 mg·kg^−1^·d^−1^) in 24/51 (47.1%) cases. Intravenous infusion of cyclophosphamide was used in 43/51 (84.3%) patients, and 8/51 (15.7%) patients were managed with oral cyclophosphamide. Intravenous immunoglobulin, plasmapheresis, and rituximab were administered in 9 (17.6%) patients, 1 patient, and one patient, respectively.

**Figure 2:**
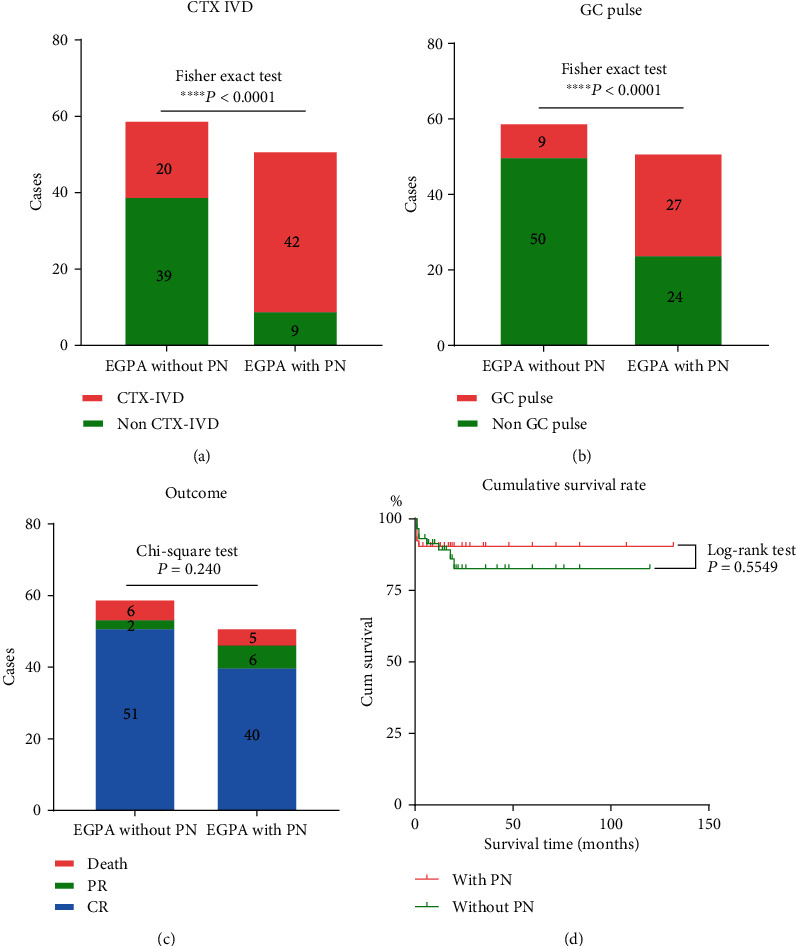
Comparison about treatment and outcome between EGPA patients with PN and without PN. (a) Comparison of intravenous infusion of cyclophosphamide; (b) comparison of initial GC pulse; (c) comparison of outcome between patients with PN and without PN; (d) comparison of cumulative survival rate between the two groups. CTX-IVD:intravenous drop of cyclophosphamide; GC pulse: glucocorticoids pulses with methylprednisolone; PR and CR: partial relief and complete remission.

Compared with patients without PN, patients with PN had more administrations of intravenous infusions of cyclophosphamide (*P* < 0.0001) (Figure 2(a)) and initial GC pulse (*P* < 0.0001) (Figure 2(b)).

### 3.10. Outcome

After a median follow-up of 18 months for patients with PN, 46 (90.2%) patients survived, and 5 (9.8%) cases died. There were no statistically significant differences in the outcome between patients with PN and without PN (Figure 2(c)). Of the five deaths, one was due to cerebral hemorrhage, one with intestinal perforation and subsequent septic shock, one was newly diagnosed with small cell lung cancer and liver metastasis, and the other two died of septic shock and multiple organ failure. The PN symptoms in about 50% of the patients did not deteriorate, and the other half improved slightly, but almost all patients did not achieve complete recovery. Regarding the cumulative survival rate between patients with PN and without PN, there was no statistical difference (Figure 2(d)).

## 4. Discussion

There are many causes of PN, including poison, malignant tumors, and systemic vasculitis [14, 15]. Among them, systemic vasculitis is one of the most important reasons. In systemic vasculitis, AAV and polyarteritis nodosa (PAN) are the two most common vasculitis with PN. The PN prevalence in PAN is up to 60%-70% [16], and in granulomatosis with polyangiitis, microscopic polyangiitis, and EGPA it is 20-25%, 40-50%, 50%-75%, respectively [17–21]. The reason why PN is more frequent in AAV and PAN is closely associated with their pathological features. The most possible pathogenesis of vasculitis-related peripheral neuropathy is inflammation of precapillary arteries in the nerves. The small arteries and venules or capillaries that supply blood to nerve fibers develop vasculitis and even necrosis, which eventually leads to nerve ischemia. Necrotic arteritis caused by AAV and PAN rarely affects the perineurial or endoneurial vessels [2, 6, 14, 22, 23]. The prevalence of PN in EGPA in our study was 46.4%, which was slightly lower compared with previous studies. The possible reason may be that different studies may have slightly different diagnostic criteria for EGPA with PN. Our diagnostic criteria were generally strict, which required not only subjective symptoms of PN, such as numbness, pain, or muscle weakness, but also critical objective examinations, such as electromyography or neuropathology, and the final diagnosis was jointly determined by the rheumatologist and neurologist.

Our study demonstrated that PN in EGPA primarily affected lower extremity, and this was supported by a recent study which revealed that peroneal nerve involvement was the most frequent and severe in EGPA-related PN [24]. PN in EGPA patients was characterized by multiple peripheral neuropathy, chronic onset, and predominantly paresthesia. In contrast to that, PAN-associated PN is often characterized by mononeuritis multiplex, acute or subacute onset, and muscle weakness [25]. In our study, the prevalence of multiple peripheral neuropathy was higher than mononeuritis multiplex, which was different from previous studies [2, 6, 15]. The possible pathogenic mechanisms are that EGPA primarily involves small vessels that supply terminal nerves, but PAN mainly affects moderate vessels that supply slightly larger nerves. So theoretically, EGPA with PN should be dominated by multiple peripheral neuropathy, and PAN-related PN should be dominated by mononeuritis multiplex. Furthermore, we observed in mononeuritis multiplex, cutaneous vasculitis and renal involvement were more frequent compared with multiple peripheral neuropathy, indicating mononeuritis multiplex was much more significantly vasculitis-mediated lesion compared with multiple peripheral neuropathy.

The study also analyzed whether PN influenced the clinical spectrum of EGPA and the associated factors for PN. According to the data, EGPA patients with PN were more prone to weight loss, arthritis or joint pain, faster ESR, and higher CRP and RF, but they did not show a significant difference about the involvement of vital organs, including the lung, kidney, CNS, heart, or digestive system, which also suggested a reason why PN did not cause poor prognosis. PN typically occurs in the early phase of EGPA [19], mainly presenting with severe inflammation, but relatively less involving other important organs. MPO-ANCA positive ratio of patients with PN was significantly higher than that without PN, but the eosinophil level in blood had no significant difference between the two groups, which was consistent with other studies [2, 8]. This suggested that PN in EGPA may be caused by ANCA-mediated vasculitis.

Multivariate logistic regression analysis demonstrated baseline ANCA positivity, arthritis or joint pain, and higher BVAS were independent associated factors for the development of PN in EGPA. Furthermore, we found the EGPA patients with BVAS ≥ 15 at baseline were more likely to develop PN. Therefore, when above factors are present in patients with EGPA, attention should be paid to whether to have peripheral nervous system involvement and further objective examination of peripheral nerves should be done.

In terms of treatment, more aggressive treatment was used to induce remission because patients with PN tended to have higher BVAS and more obvious inflammation. The primary therapy was GC pulse and high-dose intravenous infusion of cyclophosphamide, which helped achieve high clinical remission and was consistent with previous studies [26–28]. For severe refractory patients, biological agents such as rituximab, intravenous immunoglobulin, and plasma exchange were tried, and some patients showed good response during follow-up. Literature reported that rituximab was effective for some severe refractory EGPA and could improve the disease remission rate and reduce GC dosage [29, 30]; however, the studies were relatively small-scale and larger-scale randomized clinical trials in the future are warranted .

Regarding the prognosis and outcome, BVAS and the proportion of FFS ≥ 1 in patients with PN were higher than those in the control group, suggesting that patients with PN had more adverse prognostic factors. However, the final results show that regardless of whether the patients had PN, the long-term survival was similar. Considering that the proportion of PN with GC pulse and cyclophosphamide intravenous infusion was significantly higher than patients without PN, it was more likely that the selection of therapy for different groups contributed to no differences in outcome. However, we observed that neuropathy recovered slowly, and many patients had neurological sequelae, especially for badly ill patients who were not treated timely, which significantly affected their future life quality. Similarly, a study enrolling 55 EGPA patients with a median follow-up time of 75 months, evaluated the overall disability of peripheral nerve involvement in EGPA. They found the early treatment response generally was better, but long-term improvement of PN slowed down and all patients had nerve damage and disability to some extent. Especially for those patients with more severe neurological involvement, despite some improvement, they were also more likely to relapse [31].

There are still several limitations in the research. This study was retrospectively designed. Some relevant follow-up data could not be obtained. For the outcome of PN, we lacked the detailed data, because it was difficult to judge whether the PN improved and it was not objective only based on patients' own feelings unless each patient performed the electromyography. Additionally, even though the patients included in the research were from all over the country, the study was conducted in a single-center hospital and may not be a good representation for larger population with the disease.

## 5. Conclusion

PN, especially distal symmetric multiple peripheral neuropathy, is one of the most common manifestations of EGPA in China, which usually has chronic onset with paresthesia. As PN develops, it frequently involves the lower extremities, presenting with muscle weakness. ANCA positivity, arthritis or joint pain, and higher BVAS are the independent associated factors of PN in EGPA patients. Compared with multiple peripheral neuropathy, patients with mononeuritis multiplex more easily develop cutaneous vasculitis and renal involvement. GC pulse and intravenous infusion of cyclophosphamide are the most classic treatment. Although PN does not indicate poor prognosis, without prompt intervention, the neurological lesions may worsen and cause severe sequelae. Therefore, early detection, diagnosis, and intervention may improve the patients' quality of life .

## Figures and Tables

**Table 1:** Characteristics of PN in EGPA.

Type	Number	Ratio (%)
Paresthesia	42	82
Numbness	40	95
Pain	17	40
Motion abnormality	17	33
Muscle weakness	17	100
Foot drop	1	6
Wrist drop	2	12
Onset characteristics		
Subacute	16	31
Chronic	35	69
Lesion distribution		
Upper limbs	3	6
Lower limbs	22	43
Upper and lower limbs	26	51

**Table 2:** Comparison of baseline features between EGPA patients with PN and without PN.

Characteristics	With PN *n* = 51	Without PN *n* = 59	*P* value
*Demographics*			
Age (year, x ± S)	47±12.9	43±14.0	0.096
Gender (male/female, n)	27/24	33/26	0.753
Time from allergy to EGPA diagnosis (month), median (IQR)	24(3,84)	24(6,62)	0.942
Disease duration(month), median (IQR)	7(2,19)	4(2,15)	0.308
Time from initial symptoms to EGPA diagnosis (month), median (IQR)	12(0,48)	6 (0,42)	0.423
*Clinical manifestation [n (%)]*			
Weight loss	25(49.0)	16(27.1)	0.018^∗^
Fever	24(47.1)	21(35.6)	0.223
Arthritis or joint pain	12(23.5)	5(8.5)	0.029^∗^
Myalgia	14(27.5)	8(13.6)	0.069
Allergic rhinitis	15(29.4)	25(42.4)	0.159
Asthma	40(78.4)	45(76.3)	0.787
Cutaneous vasculitis	26(51.0)	33(55.9)	0.604
Renal involvement	16(31.4)	12(20.3)	0.185
Digestive tract involvement	20(39.2)	16(27.1)	0.178
CNS involvement	11(21.6)	8(13.6)	0.268
Heart involvement	20(39.2)	20(33.9)	0.563
Ear involvement	8(15.7)	6(10.2)	0.387
Sinusitis	29(56.9)	35(59.3)	0.794
Eos count (×10^9^/L), median (IQR)	3.3(1.3,9.1)	2.7(1.3,5.8)	0.827
*Clinical score*			
BVAS [recent 4 weeks, median (IQR)]	18(14,22)	12(8,16)	<0.0001^∗^
FFS ≥1[n (%)]	31(60.8)	25(42.4)	0.054

PN: peripheral neuropathy; CNS: Central Nervous System; Eos: eosinophil; BVAS: Birmingham Vasculitis Activity Score; FFS: five factor score; ^∗^*P* < 0.05.

**Table 3:** Comparison of two subtypes of peripheral neuropathy in EGPA.

Characteristics	Multiple peripheral neuropathy(*n* = 33)	Mononeuritis multiplex(*n* = 18)	*P* value
*Demographics*			
Age (year, x ± S)	49±12.9	45±12.8	0.404
Gender (male/female, n)	17/16	10/8	0.782
Time from allergy to EGPA diagnosis (month), median (IQR)	24(0, 82.5)	22(4.8, 150)	0.593
Disease duration (month), median (IQR)	6(2,18.5)	10.5(2.8, 19.3)	0.453
Time from initial symptoms to EGPA diagnosis (month), median (IQR)	12(0,48)	15(0, 51)	0.96
*Clinical manifestation[n (%)]*			
Weight loss	17(51.5)	8(44.4)	0.629
Fever	17(51.5)	7(38.9)	0.388
Arthritis or joint pain	9(27.3)	3(16.7)	0.393
Myalgia	7(21.2)	7(38.9)	0.176
Allergic rhinitis	9(27.3)	6(33.3)	0.650
Asthma	24(72.7)	16(88.9)	0.180
Cutaneous vasculitis	12(36.4)	14(77.8)	0.005^∗^
Renal involvement	7(21.2)	9(50.0)	0.034^∗^
Digestive tract involvement	12(36.4)	8(44.4)	0.572
CNS involvement	7(21.2)	4(22.2)	1.000
Heart involvement	14(42.4)	6(33.3)	0.525
Ear involvement	5(15.2)	3(16.7)	1.000
Sinusitis	19(57.6)	10(55.6)	0.889
MPO-ANCA	7(21.2)	5(27.8)	0.597
PR3-ANCA	2(6.1)	0(0)	0.287
Eos count (×10^9^/L), median (IQR)	2.93(0.43,7.42)	4.40(2.41,9.45)	0.128
Eos%, median (IQR)	22.8(9.3,47.8)	41.0(22.6,49.8)	0.161
*Clinical score*			
BVAS (recent 4 weeks,x±S)	17.82 ±5.587	21 ±6.808	0.078
FFS ≥1 [n (%)]	22(66.7)	9(50)	0.244

PN: peripheral neuropathy; CNS: Central Nervous System; MPO: myeloperoxidase; ANCA: anti-neutrophil cytoplasmic antibody; PR3: protease 3; Eos: eosinophil; BVAS: Birmingham Vasculitis Activity Score; FFS: five factor score; ^∗^*P* < 0.05.

**Table 4:** Univariate and multivariate logistic regression analysis for EGPA patients with PN.

Variable	Univariate (unadjusted)		Multivariate (adjusted)	
	OR (95% CI)	*P* value	OR (95% CI)	*P* value
ANCA positivity	5.744 (1.520,21.707)	0.010^∗^	4.387(1.030,18.683)	0.045^∗^
Arthritis/joint pain	3.323 (1.082,10.201)	0.036^∗^	3.807(1.038,13.969)	0.044^∗^
BVAS	1.177 (1.091,1.269)	<0.0001^∗^	1.117(1.025,1.217)	0.012^∗^
Weight loss	2.584 (1.168,5.718)	0.019^∗^	2.549(0.948,6.851)	0.064
FFS≥1	2.108 (0.983,4.522)	0.056	1.863(0.709,4.894)	0.207
CRP(mg/L)	1.005 (0.998,1.012)	0.186	0.999(0.990,1.008)	0.882

PN: peripheral neuropathy; ANCA: anti-neutrophil cytoplasmic antibody; BVAS: Birmingham Vasculitis Activity Score; FFS: five factor score; CRP: C-reactive protein; ^∗^*P* < 0.05.

**Table 5:** Multivariate linear regression analysis for survival time of EGPA patients.

Variable	Unstandardized *β* (95% CI)	Standardized *β*	*P* value
Age at onset	-0.498 (-0.940, -0.056)	-0.296	0.028^∗^
Gender	12.958 (1.512, 24.405)	0.280	0.027^∗^
Time from allergy to EGPA diagnosis	0.045 (-0.015, 0.105)	0.189	0.139
Peripheral neuropathy	10.390 (-1.501, 22.281)	0.225	0.085
Renal involvement	6.404 (-9.415, 22.223)	0.105	0.420
Heart involvement	-18.974 (-30.638, -7.309)	-0.396	0.002^∗^
Digestive system involvement	2.078 (-10.607, 14.762)	0.042	0.743
ANCA positivity	-10.557 (-30.549, 9.435)	-0.139	0.294
Ear involvement	10.896 (-3.796, 25.588)	0.185	0.142

ANCA: antineutrophil cytoplasmic antibody; ∗*P* < 0.05.

## Data Availability

The dataset included in this paper is available from the corresponding author on reasonable request, and with appropriate additional ethical approvals, when necessary.

## Supplementary Materials

Supplementary Materials Figure S1: the receiver operating characteristic (ROC) curve for BVAS predicting PN in EGPA.
