# Supplementary material for: Clinical Characteristics of Peripheral Neuropathy in Eosinophilic Granulomatosis with Polyangiitis: A Retrospective Single-Center Study in China
Source: J Immunol Res. 2020 Jul 4;2020:3530768. doi: 10.1155/2020/3530768 (PMC7355369; doi:10.1155/2020/3530768)
Supplement: Supplementary Materials — Figure S1: the receiver operating characteristic (ROC) curve for BVAS predicting PN in EGPA. [file 3530768.f1.pdf]

Supplementary material

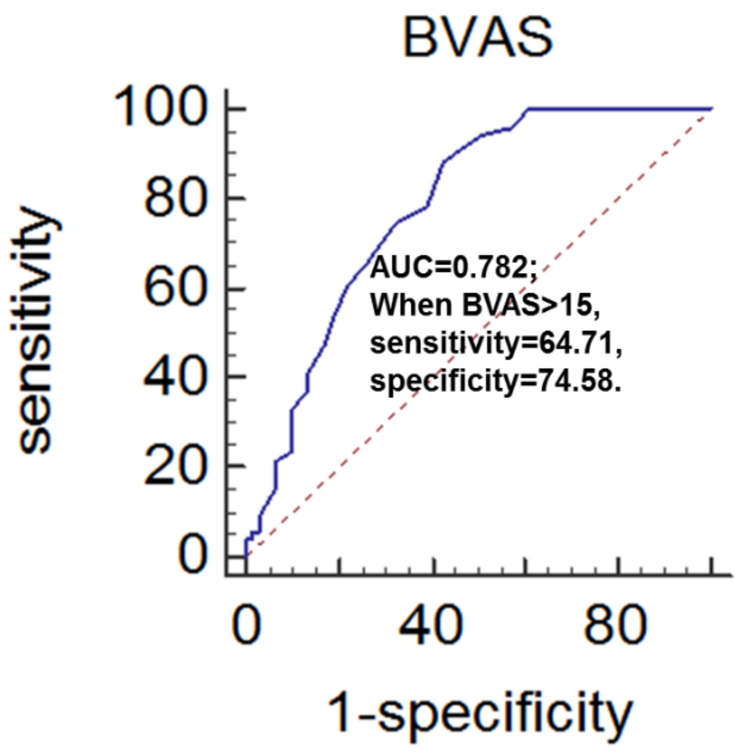

Figure S1. The receiver operating characteristic (ROC) curve for BVAS predicting PN in EGPA.
